# Changes in Bulk and Rhizosphere Soil Microbial Diversity Communities of Native Quinoa Due to the Monocropping in the Peruvian Central Andes

**DOI:** 10.3390/microorganisms11081926

**Published:** 2023-07-28

**Authors:** Richard Estrada, Roberto Cosme, Tatiana Porras, Auristela Reynoso, Constatino Calderon, Carlos I. Arbizu, Gregorio J. Arone

**Affiliations:** 1 Dirección de Desarrollo Tecnológico Agrario, Instituto Nacional de Innovación Agraria (INIA), Av. La Molina 1981, Lima 15024, Peru; genomica@inia.gob.pe (R.E.); aporrasvalencia@gmail.com (T.P.); auristela.reynoso@gmail.com (A.R.); 2 Facultad de Agronomía, Universidad Nacional Agraria la Molina (UNALM), Av. La Molina s/n, Lima 15024, Peru; 3 Facultad de Ingeniería y Ciencias Agrarias, Universidad Nacional Toribio Rodríguez de Mendoza de Amazonas (UNTRM), Cl. Higos Urco 342, Chachapoyas 01001, Peru; 4 Facultad de Ingeniería, Universidad Nacional de Barranca (UNAB), Av. Toribio Luzuriaga 376, Lima 15169, Peru; garone@unab.edu.pe

**Keywords:** microbial diversity, ITS/16S sequencing, rhizosphere and bulk soil, NGS

## Abstract

Quinoa (*Chenopodium quinoa*) is a highly nutritious crop that is resistant to adverse conditions. Due to the considerable increase in its commercial production in Andean soils, the plant is suffering the negative effects of monocropping, which reduces its yield. We used for the first time a high-throughput Illumina MiSeq sequencing approach to explore the composition, diversity, and functions of fungal and bacterial communities of the bulk and rhizosphere in soils of native *C. quinoa* affected by monocropping in the central Andes of Peru. The results showed that the bacterial and fungal community structure among the treatments was significantly changed by the monocropping and the types of soil (rhizosphere and bulk). Also, in soils subjected to monocropping, there was an increase in Actinobacteria and a decrease in Proteobacteria, and the reduction in the presence of Ascomycota and the increase in Basidiomycota. By alpha-diversity indices, lower values of bacteria and fungi were observed in the monoculture option compared to the soil not affected by monocropping, and sometimes significant differences were found between both. We detected differentially abundant phytopathogenic fungi and bacteria with growth-stimulating effects on plants. Also, we denoted a decrease in the abundance of the functional predictions in bacteria in the monocropped soils. This research will serve as a starting point to explore the importance and effects of microorganisms in degraded soils and their impact on the growth and quality of quinoa crops.

## 1. Introduction

Microbial biodiversity affects the productivity and stability of agroecosystems, so its management is agriculturally and environmentally important [1]. The causal relationships between microbial composition, diversity and abundance, and sustained soil fertility are still unclear [2]. Soil organisms constitute the axis for the functioning of the ecosystem, whose knowledge from the taxonomic perspective and function still remains poorly known [3]; in the same way, the role of biodiversity in the regulation of multiple ecosystem functions, thus limiting the possibility of predicting the loss of biodiversity and its effect on human wellbeing and the sustainability of the ecosystem [4].

The anthropic intervention, no matter how moderate, causes changes in the structure of the bacterial community and its metabolic activity in soils [5]. The components of the whole bacterial soil microbiome perform different functions, so their abundance occurs depending on the state of the soil but also some, in turn, several functions [6]. Sequencing is a fundamental tool in agricultural soils for understanding the composition and function of microorganisms present in the soil, as well as their role in plant nutrition [7].

*Chenopodium quinoa* (quinoa) is a crop that has gained popularity in recent years due to its high nutritional value and its ability to grow under adverse conditions [8,9]. Quinoa has become an important export product for many Andean countries (Peru, Bolivia, and Ecuador), where Peru has become the world’s largest producer and exporter of quinoa since 2014 [10]. While the demand for quinoa is an opportunity for Andean farmers, it is also important to address the various challenges facing the farmer to ensure that they can sustainably benefit from the global expansion of quinoa [11]. In general, a challenge that farmers face is the damage to the land resulting from monocropping, which could lead to an unfavorable impact on the production of this crop. While engaging in uninterrupted monocropping, plants of quinoa emit the same exudates repeatedly over numerous years. This occurrence sometimes results in colonization and infection by particular beneficial or harmful microorganisms that are able to utilize these substances.

Despite numerous research studies demonstrating alterations in rhizosphere soil microbial structure caused by continuous cultivation, there is a lack of knowledge of the impact of quinoa monoculture on the microbial diversity of rhizosphere soil and bulk soil. The objective of this research was to use the combination of 16S rDNA and internal transcribed spacer (ITS) sequencing techniques of fungi using MiSeq to show alterations in microbial diversity and community structure of rhizosphere soil and bulk soil of quinoa in response to a five-year continuous monoculture system.

Analysis of the diversity of quinoa soil microorganisms will identify the presence of pathogens and establish the changes in microbial diversity present in the soil under monoculture, which will help design strategies to improve soil fertility and increase quinoa yields.

## 2. Materials and Methods

### 2.1. Field Site and Sampling

**Figure 1:**
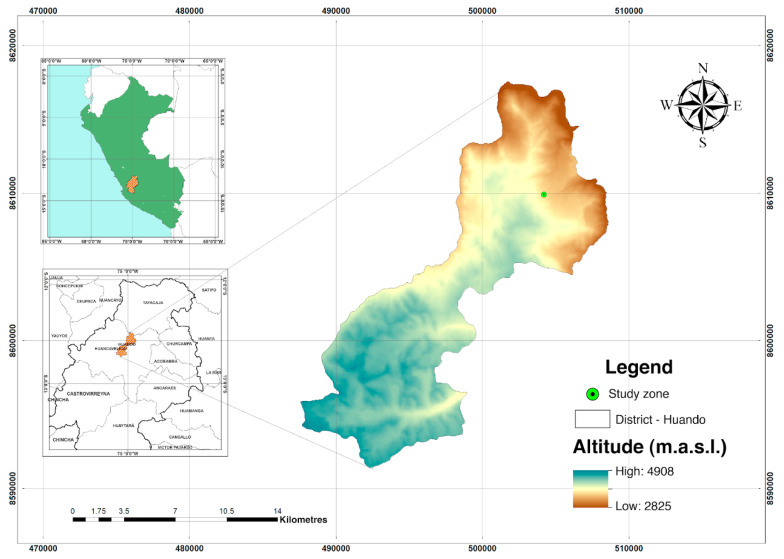
Map of the location of the two farms considered in this study.

The field experiment was conducted between December 2016 and August 2017 in the district of Huando, Huancavelica, Peru, located between the parallels 12°33′35.04″ S and 74°57′12.17″ W at an altitude of 3520 m.a.s.l. (Figure 1). Two farms were selected, one of fertile soil (FS) and another of degraded soil (DS). The FS was not cultivated for approximately 10 years, while the DS was in intensive use with monocropping for 5 years, with the use of pesticides, synthetic fertilizers, and burning of crop residues, thus affecting its productive capacity. In the two farms, the quinoa “Hualhuas” variety, with a vegetative period of six months, was cultivated. No fertilizer was used because the study was aimed at comparing the native population of bacteria and fungi from the two farms (FS and DS) through massive sequencing. The quinoa plants in both farms were sampled at 50% flowering, from which the bulk and rhizospheric soils were taken. For each plant, carefully dug up, soil farther than 1.5 cm from the roots was collected and considered as bulk soil. Then, we carefully removed the soil 5 mm away from the roots with a brush and tweezers. Soil adjacent to the root segments, at 1–5 mm from the root surface, was shaken off and defined as rhizosphere soil [12]. The soil samples were collected in triplicate depending on soil compartments (rhizosphere and bulk soil) and type of land use (with or without monocropping), and 12 soil samples were collected in total. Then, we defined four kinds of soil: degraded bulk soil (DBS), fertile bulk soil (FBS), degraded rhizosphere soil (DRS), and fertile rhizosphere soil (FRS). All soil samples were placed into sterile plastic bags, placed in an ice box, and transported to the laboratory. After passing through a 2 mm sieve, each sample was divided into two subsamples: one portion was air-dried for soil characteristic analysis, and the remainder was stored at −80 °C for DNA extraction.

### 2.2. Soil Biogeochemical Properties

The physical and chemical analysis of the soil was carried out in the Soil, Plant, Water and Fertilizer Analysis Laboratory—LASPAF of the Faculty of Agronomy, La Molina National Agrarian University (UNALM for its acronym in Spanish), Lima, Peru, where the following characteristics were analyzed: (i) percentage of sand, silt, and clay (hydrometer method), (ii) pH (potentiometer, 1:1 water/soil), (iii) electrical conductivity (conductivity meter, aqueous extract 1:1 soil/water), (iv) free carbonates (gasovolumetric), (v) organic matter (Walkley and Black), (vi) phosphorus (Olsen modified) and available potassium (flame photometer, extraction with ammonium acetate, pH 7), (vii) CEC (ammonium acetate, pH 7), and (viii) exchangeable cations (atomic absorption). The analyses were carried out following the LASPAF protocol and according to the soil and water analysis procedures manual for irrigation purposes.

### 2.3. DNA Extraction and Illumina MiSeq Sequencing

The DNA was extracted from 250 mg of soil sample with three repetitions, using the PowerSoil^®^ extraction kit (MO Bio labs, Carlsbad, CA, USA) and according to the procedure manual described by the manufacturer. The extraction was carried out aseptically, and the extracted DNA was stored at –20 °C. The concentration was quantified using the Qubit^®^ 3.0 Fluorometer (Invitrogen, Life Technologies, Van Allen Way, Carlsbad, CA, USA). Once the quality and quantity of the metagenomic DNA were verified, they were sent to the Laboratory of the Foundation for the Promotion of Health and Biomedical Research of the Valencian Community (Fisabio, Valencia, Spain) for amplification and sequencing on the Illumina MiSeq platform. They were sequenced in the MiSeq (Illumina) equipment using the V3–V4 hypervariable region of the 16S rRNA gene and ITS gene. Amplicons of approximately 460 bp in length were achieved using the following primers: Illumina_16S_341F (5′-TCGTCGGCAGCGTCAGATGTGTATAAGAGACAGCCTACGGGNGGCWGCAG-3′) and Illumina_16S_805R (5′-GTCTCGTGGGCTCGGAGATGTGTATAAGAGACAGGACTACHVGGGTATCTAATCC-3′) [13,14,15]. ITS region was amplified using the primer set ITS3_KYO2 (5′-GATGAAGAACGYAGYRAA-3′) and ITS4_KYO3 (5′-CTBTTVCCKCTTCACTCG-3′) [16,17] for fungi. To verify the size of PCR-enriched fragments, the size distribution was visualized on an Agilent Technologies 2100 Bioanalyser using a DNA 1000 chip. After size verification, the libraries were sequenced using a 2 × 300 bp paired-end run (MiSeq Reagent Kit, v. 3 (MS-102-3001)) on a MiSeq (Illumina, San Diego, CA, USA) instrument according to instructions of the manufacturer (Illumina).

### 2.4. Processing of 16S/ITS Sequences and Taxonomic Attribution

The Quantitative Insights Into Microbial Ecology microbiome analysis pipeline [18] was used to prepare and analyze the sequencing data. The DADA2 v.1.18 workflow [19] was used to process the fastQ paired-end files and create the amplicon sequence variants (ASVs). The quality filtering, trimming, and denoising of the forward and reverse reads were performed before they were merged into the ASVs. To characterize the fungal community, the q2-itsxpress plugin was used to quality filter and trim the ITS region of the sequences before processing them with DADA2. To reduce the potential for spurious ASVs, any unique sequences with a total abundance lower than 10 reads across all samples were filtered out. Taxonomic assignment of “representative” sequences was obtained by using the QIIME2 embedded naïve Bayes fitted classifier, pre-trained on the most recent Silvareference database v.138.1 for bacterial, and UNITE 7.2 database for fungal [20]. ASV tables were filtered to remove unidentified and unwanted phyla (i.e., cyanobacteria/chloroplasts) in bacteria. High-quality filtered sequences were aligned through the integrated MAFFT aligner, while rooted and unrooted 16S phylogenetic trees were constructed using the QIIME2 phylogenetic module with the FastTree algorithm.

### 2.5. Statistics Analysis

The potential effects of read sampling depth on microbial diversity calculations were evaluated by the examination of the rarefaction curves through the “diversity” QIIME module. Subsequently, several alpha-diversity measures (Shannon’s diversity index, number of observed features, Faith’s phylogenetic diversity) were computed. Statistical analyses of alpha diversity and beta diversity were performed on Qiime2-2023.1 using the “diversity alpha-group-significance” command with the pairwise Kruskal–Wallis methods. The principal coordinate analysis (PCoA) with the Bray–Curtis dissimilarity index was used to visualize differences in community composition among the groups. Beta-diversity changes among samples were tested by permutational two-way MANOVA (PerMANOVA) using the *adonis* function of vegan package and 999 permutations in R. Also, we performed the Kruskal–Wallis H test to identify differences in bacterial and fungal phylum abundance and Spearman correlation between alpha diversity in physicochemical properties in R. Furthermore, the DESeq2 package [21] in R was employed to apply a negative binomial model approach in order to detect dissimilarities in the microbial population at the genus level among groups, with a significance level set at *p* < 0.05. Finally, the resulting ASVs were assigned to their functional groups based on PICRUSt [22] and the FUNGuild tool [23] for bacteria and fungi, respectively. The top functional profiles predicted were normalized and visualized using a heatmap that was constructed using the function *heatmap.2* available in the ggplot package.

## 3. Results

### 3.1. Sequencing Results and Quality Control

In total, 12 input libraries were subjected to ITS2 sequencing, resulting in 1,460,656 raw reads. Additionally, 16S sequencing of the same number of input libraries yielded a total of 1,167,470 raw reads. Rarefaction curves based on the comparison of ASV abundance and the number of sequences analyzed (Supplementary Figures S1 and S2) tended to reach a saturation plateau, thus demonstrating that the analyses were representative of the communities under investigation.

### 3.2. Alpha/Beta Diversity

Alpha diversity was evaluated using two-way ANOVA. The results indicated that the kind of soil had a significant impact on the alpha diversity of bacteria but not on observed ASVs or phylogenetic diversity (Faith pd). Monocropping did not show any significant effects on any of the alpha-diversity indices for bacteria. Similarly, the kind of soil was found to have a significant effect on the Shannon index and Faith pd of fungi but not on observed ASVs. Monocropping showed significance only in the observed ASVs for fungi. However, the interaction between the kind of soil and monocropping did not result in any significant effects on any index of alpha diversity for either bacteria or fungi.

**Figure 2:**
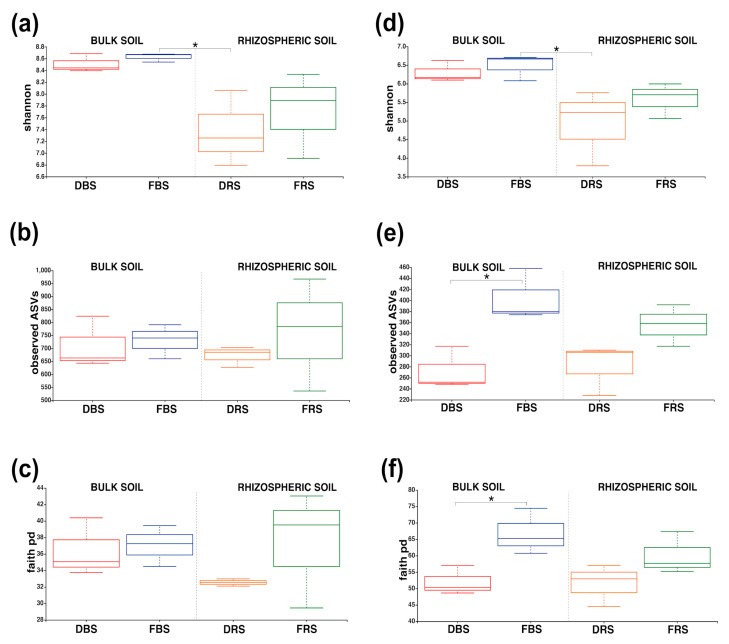
The alpha-diversity analysis was performed on soil samples collected from DBS, FBS, DRS, and FRS. The analysis was conducted separately for bacterial and fungal organisms, and three measures were used: Shannon’s diversity index for bacteria (**a**) and fungi (**d**), observed features for bacteria (**b**) and fungi (**e**), and Faith’s phylogenetic diversity for bacteria (**c**) and fungi (**f**). Any statistically significant differences among the soil samples are denoted with an asterisk (*), with a significance level of *p* < 0.05.

Also, the result of the alpha-diversity metric for bacteria and fungi showed the highest indices of average Shannon for FBS (H_B-FBS_ = 8.63, H_F-DBS_ = 6.5) (Figure 2a,d). The qualitative evaluation of community richness by the observed ASVs metric indicates that FRS (O_B-FRS_ = 762.33) leads the bacterial diversity while FBS (O_F-FBS_ = 404) leads to fungal diversity (Figure 2b). Faith pd indicated that there is more phylogenetic diversity of bacteria in samples collected of FRS (F_B-FRS_ = 37.34) and FBS (F_B-FBS_ = 37.07), and for fungal diversity, there are significant differences between FBS (FF-FBS = 66.8) and DBS (FF-DBS = 52.02).

The results of the physicochemical analyses are shown in Table S1. Also, an analysis of Spearman connections was carried out for degraded and fertile rhizospheric soils. Figure S4b shows that for fungi, there is a correlation between some edaphic factors, such as Na cation and CE, with respect to the Shannon diversity index, while in bacteria (Figure S4a), no significant correlations were found between both factors.

**Figure 3:**
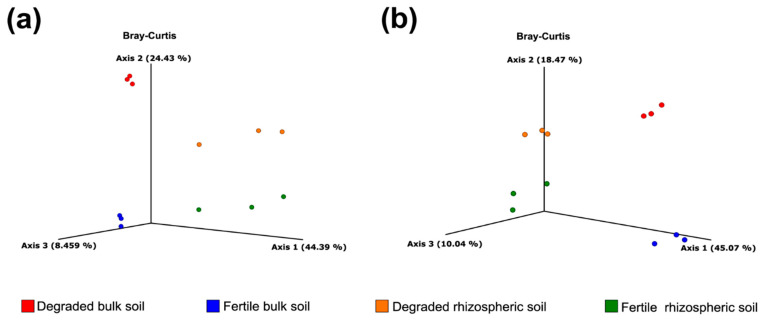
Beta-diversity measures for the samples collected (DBS, FBS, DRS, and FRS) in bacteria (**a**) and fungi (**b**). Separation of bacterial and fungal communities in the analyzed soils is shown according to a PcoA analysis based on Bray–Curtis distances. Individual points represent sequenced soil replicates.

Figure 3 presents the PCoA and reveals differences in bacterial and fungal diversity (DBS, FBS, DRS, FRS). The result revealed strong differences in community structure between them. There was a separation between the rhizosphere and bulk soil groups along axis 1 and axis 2 in Bray–Curtis PCoAs. Furthermore, PerMANOVA analysis (Table 1) indicated a significant effect of the kind of soil (bulk and rhizosphere) and monocropping (fertile and degraded) for bacterial and fungal communities. Also, we detected that the interactions of these effects were also significant in bacteria but not in fungi.

### 3.3. Taxonomic Composition of Fungal and Bacterial Communities in Rhizosphere Soil

**Figure 4:**
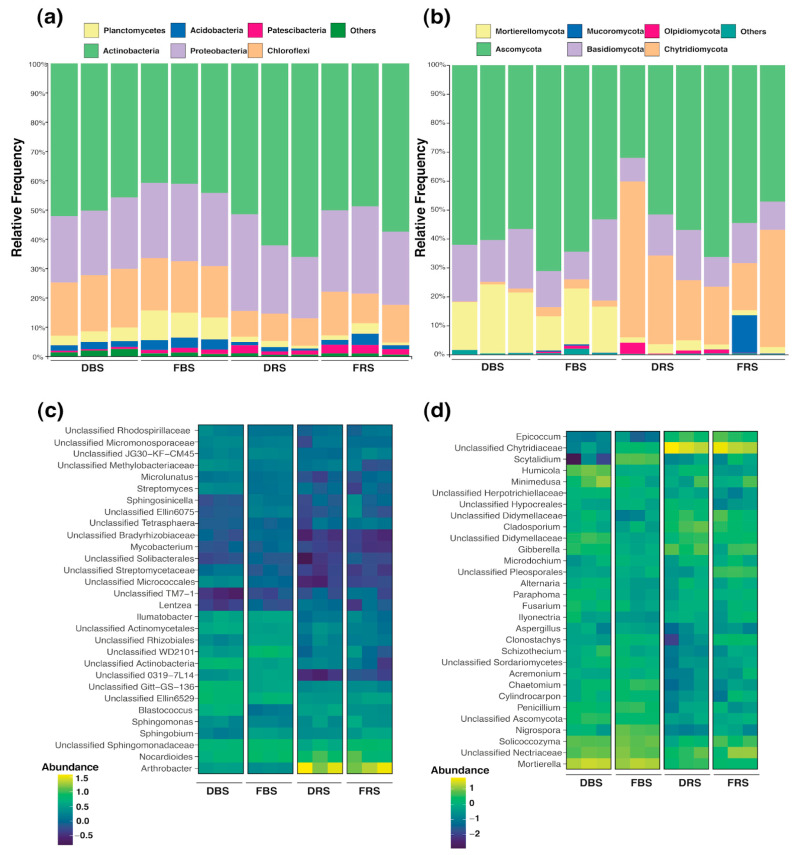
Taxonomic summaries in the bulk and rhizospheric soil at fertile and degraded soil. Relative abundance of the dominant (**a**) microbial composition at the phylum level in bacteria and (**b**) at the phylum level in fungi. Analysis of heatmap of top 30 dominant bacteria (**c**) and fungi (**d**) genera present. Relative abundance data were z-scored and normalized by row.

ASVs from 16S and ITS were classified using a 99% sequence similarity threshold against the Silva 138.1 database and UNITE database, respectively. Figure 4 indicates a graphic of taxonomic compositions for the four groups evaluated. The results present significant differences in the groups evaluated and inside them. The taxonomic composition shows that the group of bacteria is dominated by phylum Actinobacteria and Proteobacteria, and fungal groups by phylum Ascomycota and Basidiomycota.

For bacteria, there was a change in the relative abundance of the dominant phylum for soils with monocropping. In Actinobacteria, there were increases, where FRS presented 52.12%, DRS 59.9%, and FBS obtained 42.05% and 49.37% for DBS. In contrast, in Proteobacteria, there were decreases, where FRS presented 27.55% and DRS 25.77%, FBS 25.71%, and 23.06% DBS. Similar decreases were registered in the phylum Chloroflexi and Planctomycetes. The remaining phyla have less abundance in comparison to the rest (Figure 4a).

In fungi, changes in dominant phyla were also recorded in soils with monocropping. The most abundant phylum was Ascomycota, and in FRS soil, it accounted for 56.06%, while in DRS, it was 46.88%. Similarly, in FBS soil, it was 62.98%, while in FDS, it was identified as 59.73%. In Basidiomycota, there were slight increases, with FRS representing 11.28% and DRS 13.21%, as well as in FBS (16.66%) and DBS (18.18%). On the other hand, there was a significant change in the abundance of the Chytridiomycota phylum, as it obtained a greater representation in rhizospheric soils (30.35%) than in bulk soil (1.98%) and an increase from FRS (25.60%) to DRS (35.10%) was recorded, with a slight decrease in relation to FBS (2.83%) and DBS (1.13%). In Planctomycetes, there were decreases from FRS with 1.78% and DRS with 1.48%, and in FBS with 7.96% and DBS with 3.38%. In comparison to the others, the remaining phyla are less abundant (Figure 4b). Based on the comparisons, significant differences were found in the bacterial phyla: Actinobacteria, Planctomycetes, Acidobacteria, and Chloroflexi and for the fungal phyla: Chytridiomycota and Mortierellomycota (Table S4).

The heatmap of taxonomy comparison analysis at the genus level was also displayed (Figure 4c,d). The results for the bacterial community showed marked differences between rhizosphere soils and bulk soils. In particular, the genus *Arthrobacter* showed more abundance in rhizospheric soils than in bulk soil, and the genus unclassified WD2101, unclassified *Actinobacteria*, unclassified *Gitt-GS-136*, unclassified *El-lin6529* were the most abundant genera found only in bulk soil group. The degraded soil has a higher abundance of genus unclassified *Git-GS-136* and *Blastococcus*, while fertile soil has an abundance of bacteria of the unclassified *WD2101* (Figure 4c).

In the fungi group, there is a greater presence of genera such as *Mortierella*, unclassified *Nectriaceae*, *Solicoccozyma*, unclassified *Didymellaceae,* and *Gibberella*. In the DBS, there is a higher presence of fungi of the genus *Minimedusa* and *Humicola*, but in the FBS, there was a higher abundance of the genus Sistotrema. In the rhizosphere soils, there are notable differences between the two taxa. Fertile soil had an abundance of *Nectriaceae* and degraded soil for the *Cibberella* (Figure 4d).

### 3.4. Differences in Relative Abundance and Functional Diversity

**Figure 5:**
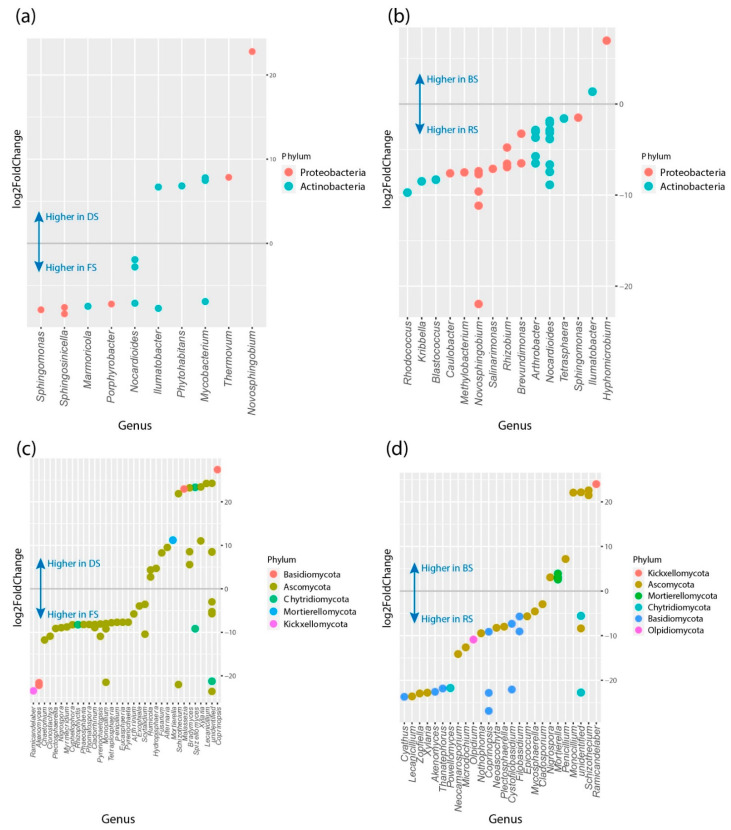
Pairwise comparison (DeSeq2 analysis) generated plots of variations of abundance ASVs between the groups (*p* < 0.05). ASVs at the genus level (*x*-axis) and phylum level (colors). In bacteria species, (**a**,**b**) negative “log2 fold change” values (*y*-axis) indicate higher abundance in fertile soil samples, and positive values indicate higher abundance in degraded soil samples. In fungal species, (**c**,**d**) negative “log2 fold change” values (*y*-axis) indicate higher abundance in degraded soil samples, and positive values indicate higher abundances in fertile soil samples ((**c**): Bulk, (**d**): Rhizosphere).

In line with these results, The DESeq2 method was utilized to pinpoint essential genera that may exhibit differential correlations with the existence or non-existence of monocropping in bulk and rhizosphere soils. Overall, a larger quantity of noteworthy bacterial and fungal genera was detected at higher occurrences in fertile soils (without monocropping) (Figure 5 and Supplementary Figure S3). For bulk soil, DESeq2 identified a fungal enrichment (log2 fold change < 0) of 26 fungal members in fertile soil, including ASVs from the phylum Ascomycota (23 ASVs) and in degraded soil (log2 fold change > 0) were identified 14 fungal members (Figure 5c). In the bacterial enrichment (log2 fold change < 0) of 15 bacterial members, phylum Actinobacteria (nine ASVs) and Proteobacteria (six ASVs) in fertile soil and for degraded soil indicate 9 ASVs where the predominant phylum was Actinobacteria (Figure 5a). In the case of rhizospheric soil, DESeq2 identified (log2 fold change < 0) 10 fungal members, and for degraded soil, 7 fungal members were recognized. The predominance phylum was Ascomycota in both types of soil (Figure 5d). The bacterial analysis of rhizospheric soil indicated only 2 ASV in comparison to degraded soil (4 ASVs) (Figure 5b).

We analyzed two groups based on metadata: soil type (bulk and rhizosphere) and treatment (degraded and fertile), respectively (Supplementary Figure S3). For fertile soil, DESeq2 identified bacterial enrichment (log2 fold change < 0) of 10 bacterial members, and in the case of degraded soil (log2 fold change > 0), a particular genus, *Novosphingobium* (Supplementary Figure S3a). The quantitative bacterial members of rhizospheric soil (log2 fold change < 0) were notably more abundant in contrast to bulk soil (log2 fold change > 0) had two ASVs (S.5b). The analysis of fungal members in soil type and treatment showed more (log2 fold change < 0) ASV from phylum Ascomycota (Supplementary Figure S3c,d).

**Figure 6:**
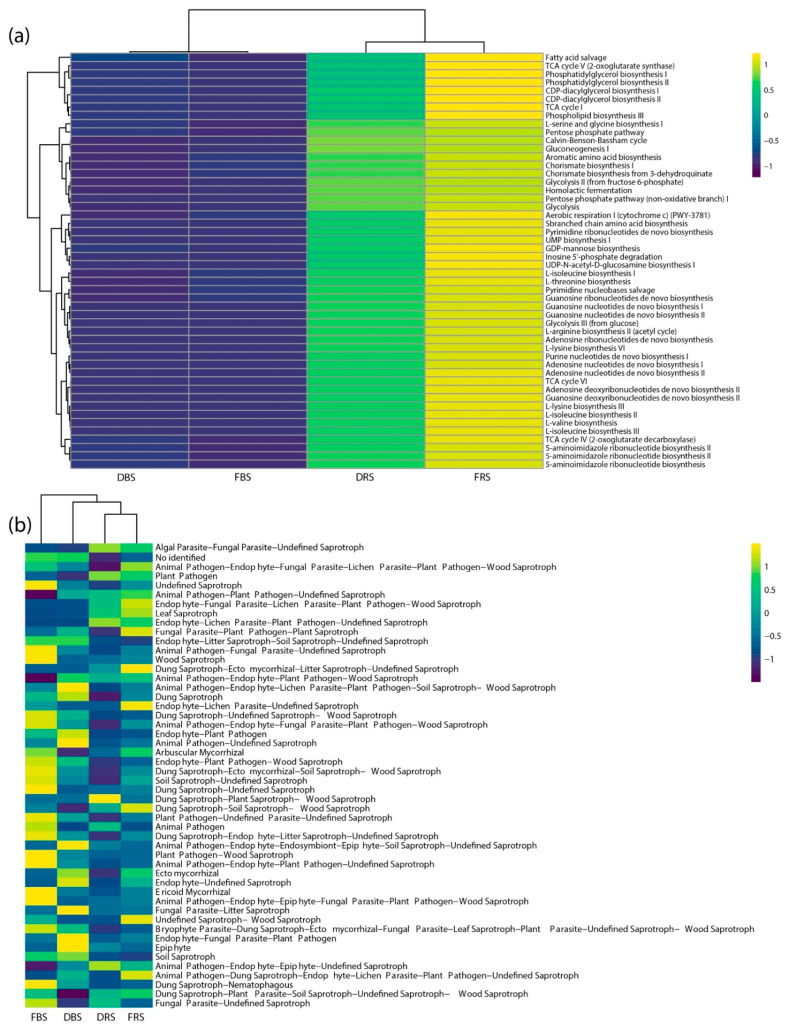
A heatmap is utilized to display the 30 top anticipated functional abundance ratios of the bacterial and fungal communities, utilizing Picrust-MetaCyc (**a**) and FUNGuild (**b**), respectively, as a basis for functional prediction analysis. To represent relative changes across the groups, the relative abundance was normalized to a Z-score.

Also, the relative abundances of pathways predicted by PICRUSt and FUNGuild revealed that the functional profiles were different among the rhizospheric and bulk soils. About the 30 MetaCyc pathways with the most abundance, it was identified that the rhizospheric groups present greater abundance compared to rhizospheric soils. Within them, we identified an overrepresentation of L-isoleucine biosynthesis II-I, L-valine biosynthesis, and branched-chain amino acid biosynthesis. In addition, a decrease in abundance in predictions is denoted in monocropping soils for rhizosphere and bulk soils (Figure 6a). On the other hand, in the top 30 profiles FUNGuild, among the important changes, we denoted an increase in the category of arbuscular mycorrhizal and ectomycorrhizal and a decrease in the pathogen plants of the FRS compared to the DRS. We also detected the highest amount of indefinite saprotroph and wood saprotroph in FBS, and a higher abundance of leaf saprotroph in FRS, compared to the others. One important issue with the FUNGILD database, in general, is the fact that some fungi do not fall exclusively into a single guild. For example, the prediction of undefined saprotroph-wood saprotroph was overrepresented in FRS (Figure 6b).

## 4. Discussion

Quinoa is an age-old plant that has the ability to endure cold, salty, and dry conditions. Additionally, it is incredibly healthy and boasts a wide range of genetic variations due to its sporadic and geographically specific cultivation throughout the Andean region. The results of this work indicated that the microbial community in the soil was affected by continuous and prolonged quinoa cultivation, as revealed by the analysis of 16S rRNA and ITS sequences using Illumina MiSeq sequencing technology. 

The soil bacterial community was dominated by Actinobacteria and Proteobacteria (Figure 2a). This profile is similar to the report in Jiangsu Province, China [24], with the exception of Chloroflexi, which was reported in the top three of the phylum for our study. The presence of a high number of bacteria from the phyla Proteobacteria and Actinobacteria in the soil is regarded as a dependable parameter for the surveillance of soil wellbeing [25]. There was an increase in Actinobacteria and a decrease in Proteobacteria in soils with monocropping. Proteobacteria could demand particular nutrients, such as nitrogen or phosphorus, to proliferate and thrive. In case these nutrients are absent in the soil, the population of Proteobacteria might reduce. It has been recorded that monocropping decreases the populations of Proteobacteria [26]. On the other hand, Actinobacteria could potentially degrade crop panicles during the harvesting of quinoa, where the panicles are scattered in the field and used as a metabolic source for microorganisms. This could be a plausible explanation for the increase in the Actinobacteria phylum.

To the best of our knowledge, there is no history of metataxonomic fungal studies of rhizosphere or bulk soil in *C. quinoa*. In our study, the soil fungal community was dominated by Ascomycota and Basidiomycota in monocropping soils (Figure 4b). Ascomycota are extensively acknowledged as fungi that decompose cellulose and have a restricted capacity to degrade lignin [27,28]. Basidiomycota have a tendency to break down stubborn lignin-containing matter and, hence, dominate the later phase of litter decomposition [29]. We identified a significant decrease in the organic matter content in rhizospheric soils with monoculture compared to the others (Table S2). Possibly the change in the percentages of Ascomycota and Basidiomycota in soils with monoculture indicated a modification in the carbon composition of the soil caused by the monoculture. This has already been identified in other crop systems [30,31,32,33]. There is a possibility that more recalcitrant C was gradually amassed, and labile C was exhausted in the soil. This results in a higher number of Basidiomycota, which decompose lignin, and fewer Ascomycota, which are sugar fungi, in monocropping soils.

A higher presence of Planctomycetes has been observed in fertile soils compared to degraded soils. Planctomycetes are a group of soil bacteria known for their ability to fix and decompose nitrogen, suggesting a possible relationship with nitrogen availability in these soils [34,35]. In fertile soils, where higher amounts of Planctomycetes are found, it is possible that there is better nitrogen availability due to the ability of these bacteria to convert nitrogenous compounds into forms usable by plants [36].

The fact that rhizosphere microbiota differ from bulk soil microbiota is well established. Our research suggests that the variation in the type of land use has a greater impact on the diversity of the fungal community compared to that of the bacterial community. Our principal coordinates analysis illustrates how extended periods of continuous cropping affect the microbial communities of both bacteria and fungi in the soil (Figure 3). This occurrence has also been detected in continuous cropping systems used for growing *Panax notoginseng* [37], peanuts [38], and in monocropping cultivation of vanilla [39].

Also, the results of the alpha-diversity indices show that there are significant differences within and between soil types: bulk and rhizospheric. The alpha-diversity plots show the lower diversity of bacteria and fungi in the degraded bulk soil versus the fertilized bulk soil, and in some cases, the differences were significant (Figure 2). This may be due to the fact that degraded soils are sources that have been used for monocropping. The damage of monocropping to soil microbiological diversity is known because if a single plant species provides a constant source of nutrients and exudes specific chemicals through its roots, it creates a homogeneous environment in the soil and favors the growth of certain species of microorganisms to the detriment of others [40]. Also, monocropping often requires the intensive use of agrochemicals, such as pesticides and fertilizers, to control diseases and pests and maintain high crop yields. These agrochemicals can have negative effects on the diversity and activity of soil microorganisms, as they can kill or inhibit certain microbial species, leading to a decrease in microbial diversity [41].

A significant decrease in potassium levels was found for DRS compared to FRS (Table S2). This variation is probably related to microbiological diversity. This also coincides with the study by Xu et al. [42], in which he mentions that potassium plays a crucial role by enriching the nutrients in the environment, thus creating an optimal and healthy environment for the growth of microorganisms. In addition, a significant difference was observed in organic matter, with a decrease in degraded rhizosphere soil (DRS) values (Table S2), thus agreeing with other studies [43,44] that indicated that an adequate supply of available organic matter can promote a significant increase in bacterial diversity, both in terms of species richness and phylogenetic diversity of microorganisms. 

The results of the correlation analysis show that electrical conductivity and the amount of Na cation have an inverse relationship with microbiological diversity (Shannon index). An optimal level of electrical conductivity and Na allows an adequate distribution of ions and nutrients in the soil, which provides a favorable environment for the growth of microorganisms, as it helps to maintain a proper balance of water and moisture in the soil, essential for survival [45,46]. Therefore, electrical conductivity has been reported as a factor that is negatively related to microbiological diversity [47].

The DESeq2 results identified notable differences between soil types, with the majority of ASVs belonging to fertile soils that included the phylum Ascomycota and Basidiomycota as the most abundant (Supplementary Figure S3c). A high abundance of *Coprinopsis* was observed in degraded soils; this genus may be more common in degraded or disturbed soils compared to fertile or undisturbed soils [48] (Supplementary Figure S3c). In addition, a significant presence of *Alternaria* and *Fusarium* has been observed. The proliferation of these phytopathogens could be due to the effects of monocropping, as they generate a decrease in diversity and changes in the soil. The use of chemicals is another factor to consider [40]. In the analyses according to soil type and treatment, degraded soils were found to have a high abundance of the genus *Novosphingobium* (Supplementary Figure S3a). Monocropping leads to the continuous use of pesticides that affect the abundance of certain bacterial genera that benefit from their components. *Novosphingobium* is a genus identified as the third most abundant after an analysis of soils that were subjected to pesticides [49,50]. On the other hand, we identified three differentially abundant *Nocardioides* ASVs and one *Porphyrobacter* ASV in soils without monocropping (Supplementary Figure S3a). *Nocardioides* has been reported as a growth-promoting genus in *Beta vulgaris* [51], similar to Porphyrobacter in *Sitobion avenae* [52].

All 30 top categories of gene function from PICRUSt were clearly more abundant in the rhizospheric soil (Figure 6a), we sampled the soils in flowering, and it can increase the functional diversity of the rhizosphere due to increased activity and exudation of plant roots during this period [53,54]. Also, we detected a decreasing abundance of functional diversity in degraded soils (with monocropping). The primary reason for the occurrence of quinoa soil sickness could be attributed to the reduction in both the abundance and diversity of bacterial communities, particularly those that are beneficial, as well as the simplification of bacterial community function, all of which are associated with long-term monocropping of quinoa. About profiles in FUNGuild, we detected a higher abundance of guild pathogens in soils with monocropping in comparison with the others. The increase in pathogens in the soil as a consequence of monocropping has already been previously reported. [39,55]. In addition, we observed a higher amount of saprotroph profiles in non-monocropping soils (Figure 6b), which are fungi that derive nutrients from the breakdown of dead organic material, typically indicating a higher level of functional diversity among fungi [56].

Understanding the diversity patterns of microbial communities for different monocropping and management systems will help clarify the relationship between the continuous planting of quinoa and soil degradation.

## 5. Conclusions

This is the inaugural study shedding light on the impact of a persistent monocropping system on the diversity and composition of soil microorganisms within *C. quinoa* plantations. The findings indicated that both monocropping and the type of soil (rhizosphere and bulk) had a significant impact on the structure of the bacterial and fungal communities. Also, in rhizosphere soil, we found significance in the negative correlations of Na and CEC with alpha diversity. Furthermore, the study discovered soil biomarkers specific to *C. quinoa* that enables the identification of bacteria and fungi with significantly varying abundance in soil samples. This research offers a valuable understanding of the underlying mechanism responsible for challenges in continuous monocropping systems and holds the potential for enhancing the productivity and quality of this crop.

## Figures and Tables

**Table 1:** Two-way PerMANOVA test parameters of the influence of kind of soil or monocropping on the bacterial and fungal community composition.

	Variable	R2	F	Pr (>F)
Bacterial	Kind of soil	0.3372	7.3989	0.001 *
	Monocropping	0.1659	3.6412	0.035 *
	Kind of soil × Monocropping	0.1323	2.9050	0.048 *
Fungal	Kind of soil	0.4111	9.3141	0.001 *
	Monocropping	0.1618	3.6651	0.011 *
	Kind of soil × Monocropping	0.0740	1.6768	0.107

(*) Implicit significance (*p* < 0.05).

## Data Availability

Raw sequence reads (SRA accession numbers SRR2467728/SRR24677301) are available in the NCBI GenBank database under BioProject accession number PRJNA974498.

## Supplementary Materials

The following supporting information can be downloaded at: https://www.mdpi.com/article/10.3390/microorganisms11081926/s1, Figure S1. Rarefaction curves of species richness showing the sequencing depth of 16S data obtained from soil. Figure S2. Rarefaction curves of species richness showing the sequencing depth of ITS data obtained from soil. Figure S3. Pairwise comparison (DeSeq2 analysis) generated plots of variations of abundance ASVs between the groups (*p* < 0.05). Figure S4. Correlation between alpha diversity index and edaphic factors. Table S1. Values of edaphic factors and alpha diversity indices of fertile (FRS) and degraded (DRS) rhizospheric soils. Table S2. Paired *t*-test on soil physicochemical variables. Different letters indicate significance at *p* < 0.05. Table S3. Kruskal-Wallis H test on the five most abundant bacterial phyla. Only those phyla that showed significant differences are reported. Table S4. Kruskal-Wallis H test on the five most abundant fungal phyla. Only those phyla that showed significant differences are reported.
