# Supplementary material for: Changes in Bulk and Rhizosphere Soil Microbial Diversity Communities of Native Quinoa Due to the Monocropping in the Peruvian Central Andes
Source: Microorganisms. 2023 Jul 28;11(8):1926. doi: 10.3390/microorganisms11081926 (PMC10458079; doi:10.3390/microorganisms11081926)
Supplement: Supplementary file 1 [file microorganisms-11-01926-s001.zip › microorganisms-2435438-supplementary/Supplementarty Figures.pdf]

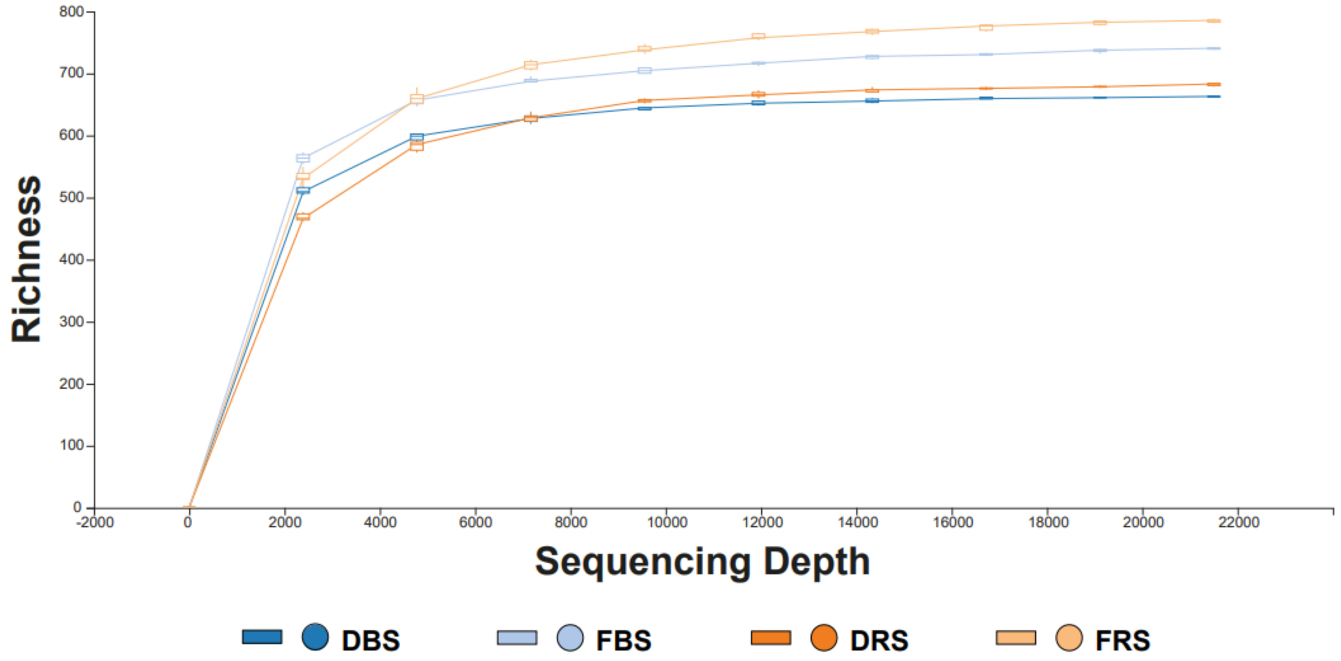

Figure S1. Rarefaction curves of species richness show the sequencing depth of 16S data obtained from soils. Different colors displayed the rarefaction curves for each kind of soil. The x-axis represents the sequencing depth in the number of reads and the y-axis is the ASV richness detected atthe species level. DBS: Degraded Bulk Soil, FBS: Fertile Bulk Soil, DRS: Degraded Rhizosphere Soil, and FRS: Fertile Rhizosphere soil.

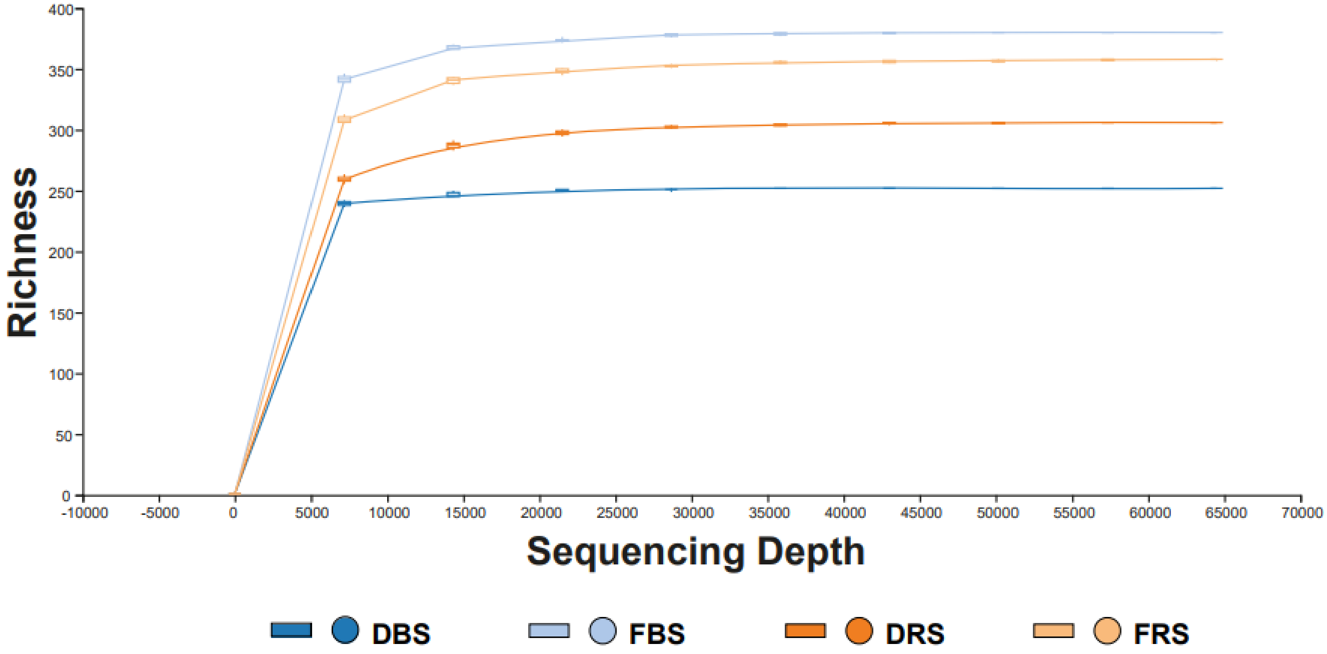

Figure S2. Rarefaction curves of species richness show the sequencing depth of ITS data obtained from soils. Different colors displayed the rarefaction curves for each kind of soil. The x-axis represents the sequencing depth in the number of reads and the y-axis is the ASV richness detected atthe species level. DBS: Degraded Bulk Soil, FBS: Fertile Bulk Soil, DRS: Degraded Rhizosphere Soil, and FRS: Fertile Rhizosphere soil.

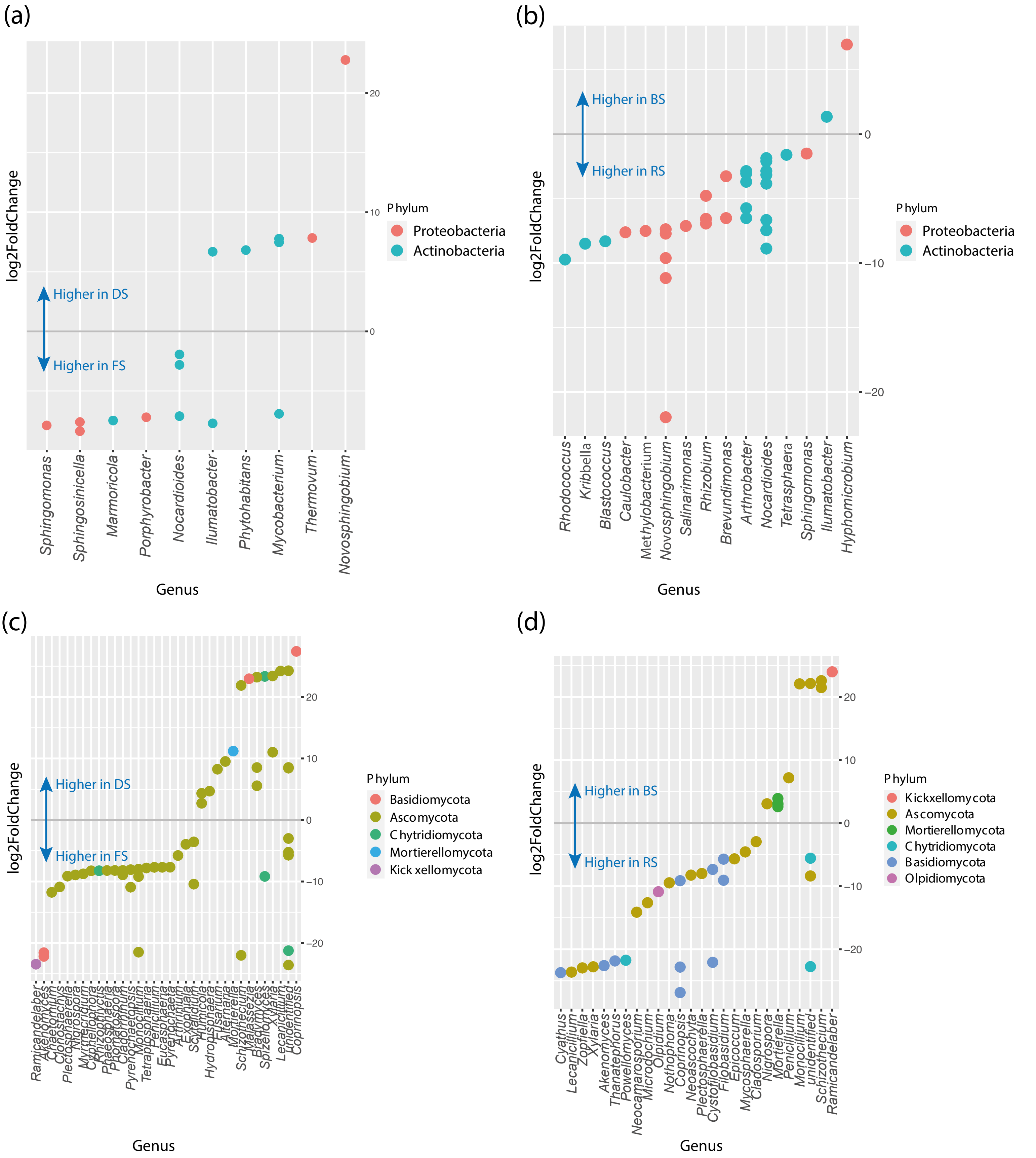

Figure S3. Pairwise comparison (DeSeq2) generated plots of variations of abundance ASVs between the groups ($p < 0.05$).

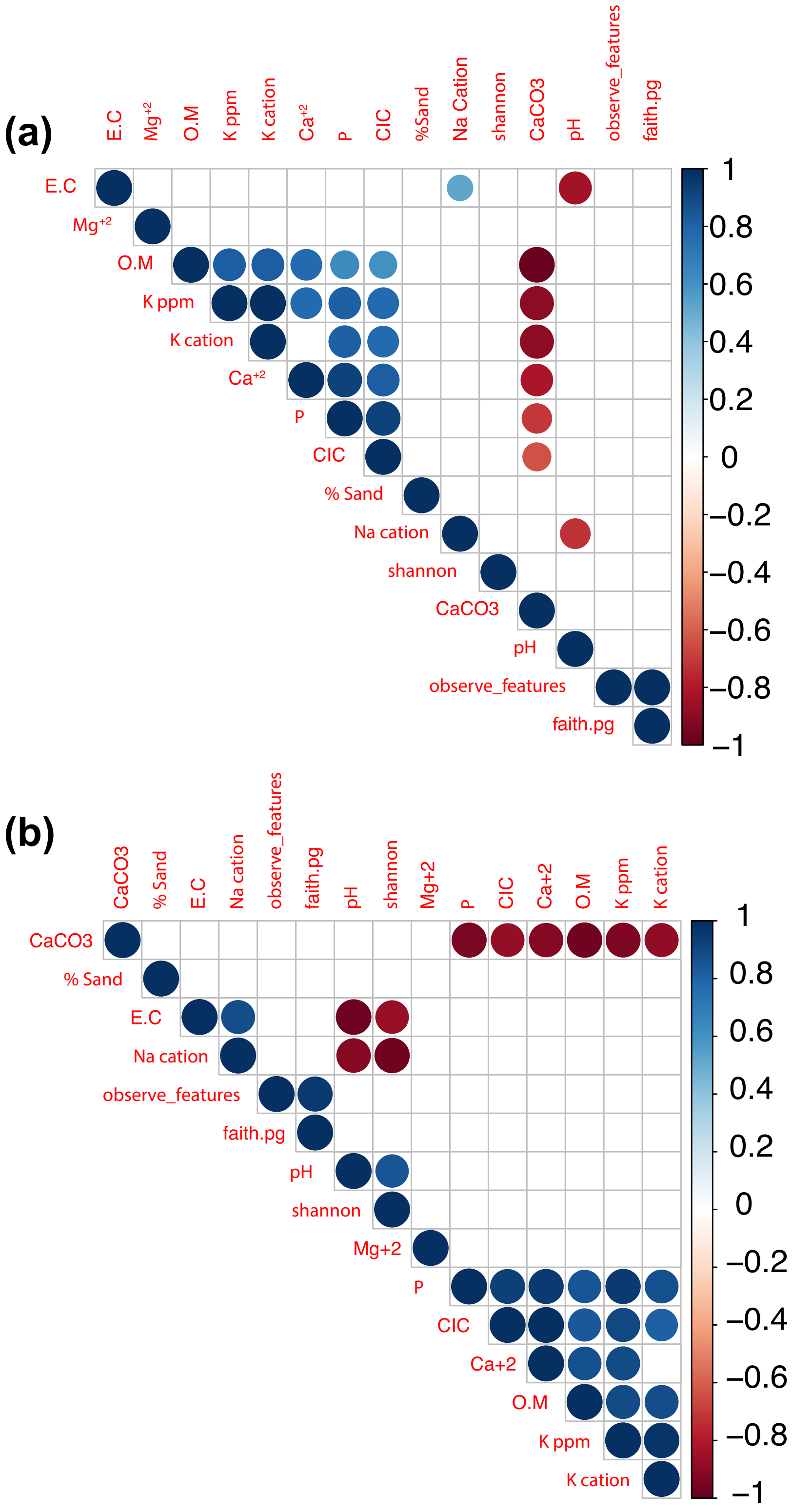

Figure S4. Correlation between alpha diversity index and edaphic factors. The diversity index of bacteria (A) and fungi (B) was correlated with 12 biotic variables. Spearman correlations were used to describe the relationships, with positive interactions shown in blue and negative interactions shown in red. The shading color and size of the circles represent the strength of the correlation on an absolute scale ranging from 0 to 1.
