# Supplementary material for: Changes in Bulk and Rhizosphere Soil Microbial Diversity Communities of Native Quinoa Due to the Monocropping in the Peruvian Central Andes
Source: Microorganisms. 2023 Jul 28;11(8):1926. doi: 10.3390/microorganisms11081926 (PMC10458079; doi:10.3390/microorganisms11081926)
Supplement: Supplementary file 1 [file microorganisms-11-01926-s001.zip › microorganisms-2435438-supplementary/Supplementarty Tables.pdf]

Sample and replicates	Edaphic factors												Shannon		Observe features		Faith PD	
	pH	E.C	CaCO3	O.M	P ppm	K ppm	CIC	Ca+2	Mg+2	K+	Na+	% Sand	B	F	B	F	B	F
**FRS R1**	8.16	0.19	10.1	2.07	8	439	27.2	23.92	1.88	1.23	0.17	40	7.890	4.1516	784	358	39.5250	57.6901
**FRS R2**	8.13	0.21	10.2	1.98	8.8	390	31.2	28.08	1.97	0.94	0.21	36	6.9104	3.9534	536	317	29.4545	55.1869
**FRS R3**	8.21	0.16	10.3	1.58	8.8	371	27.84	25.05	1.78	0.78	0.23	38	8.3321	3.5101	967	392	43.0372	67.3402
**DRS R1**	7.8	0.57	14.3	1.08	3.25	148	21.44	18.88	1.92	0.3	0.34	42	8.0614	2.6314	685	228	32.5487	44.552
**DRS R2**	8.13	0.16	14.3	0.63	4.7	190	21.6	19.09	1.82	0.47	0.23	42	7.2559	3.6263	627	306	32.1099	57.0597
**DRS R3**	8.27	0.12	16.8	0.17	3.4	165	22.08	18.21	1.88	0.32	0.15	38	6.7927	3.9894	703	310	32.9803	52.9554

Table S1. Values of edaphic factors and alpha diversity indices of fertile (FRS) and degraded (DRS) rhizospheric soild. B: bacteria, F: fungi.

Parameters	Average in FRS	Average in DRS
**pH**	8.17[a]	8.07[a]
**C.E**	0.19[a]	0.28[a]
**CaCO3**	10.2[b]	15.13[a]
**O.M**	1.88[a]	0.63[b]
**P (ppm)**	8.53[a]	13.83[b]
**K (ppm)**	400[a]	167.67[b]
**CIC**	28.75[a]	21.71[b]
**Ca+2**	25.75[a]	18.73[b]
**Mg+2**	1.88[a]	1.87[a]
**K+**	0.98[a]	0.36[b]
**Na+**	0.2[a]	0.24[a]
**% Sand**	38[a]	40.67[a]
**% Silt**	27[b]	32.33[a]
**% Clay**	35[a]	27[b]

Table S2. Paired t-test on soil physicochemical variables. Different letters indicate significance (at $p < 0.05$). FRS: rhizosphere soil, DRS: Degraded rhizosphere soil.

**BACTERIA** Phylum		p-value
Actinobacteria		
	DRS-DBS	0.1047
	FBS- DBS	0.3143
	FRS-DBS	0.8948
	FBS-DRS	0.0082*
	FRS-DRS	0.2710
	FRS-FBS	0.1234
Acidobacteria		
	DRS-DBS	0.4328
	FBS- DBS	0.2502
	FRS-DBS	0.9798
	FBS-DRS	0.0273*
	FRS-DRS	0.2725
	FRS-FBS	0.4014
Chloroflexi		
	DRS-DBS	0.0001*
	FBS- DBS	0.2636
	FRS-DBS	0.0009*
	FBS-DRS	0.0003*
	FRS-DRS	0.0563
	FRS-FBS	0.0098*
Planctomycetes		
	DRS-DBS	0.1344
	FBS- DBS	0.0025*
	FRS-DBS	0.2370
	FBS-DRS	0.0002*
	FRS-DRS	0.9739
	FRS-FBS	0.0003*

Tabla S3. Kruskal-Wallis H test was performed on the five most abundant bacterial phyla. Only those phyla that showed significant differences are reported.

**FUNGI** Phylum		p-value
Chytridiomycota		
	DRS-DBS	0.0187*
	FBS- DBS	0.9954
	FRS-DBS	0.0846
	FBS-DRS	0.0255*
	FRS-DRS	0.7078
	FRS-FBS	0.1168
Mortierellomycota		
	DRS-DBS	0.0002*
	FBS- DBS	0.2032
	FRS-DBS	0.0001*
	FBS-DRS	0.0017*
	FRS-DRS	0.9669
	FRS-FBS	0.0010*

Table S4. Kruskal-Wallis H test on the five most abudant fungal phyla. Only those phyla that showed significant differences are reported.
